# Synthesis, crystal structure and thermal decomposition pathway of bis­(iso­seleno­cyanato-κ*N*)tetra­kis­(pyridine-κ*N*)manganese(II)

**DOI:** 10.1107/S2056989023003535

**Published:** 2023-04-21

**Authors:** Christian Näther, Sebastian Mangelsen, Jan Boeckmann

**Affiliations:** a Institut für Anorganische Chemie, Universität Kiel, Max-Eyth-Strasse 2, 24118 Kiel, Germany; University of Aberdeen, United Kingdom

**Keywords:** crystal structure, nickel seleno­cyanate, discrete complex, thermal properties

## Abstract

The reaction of MnCl_2_·2H_2_O with KSeCN and pyridine in water leads to the formation of the title complex, [Mn(NCSe)_2_(C_5_H_5_N)_4_], which is isotypic to its Fe, Co, Ni, Zn and Cd analogues. In its crystal structure, discrete complexes are observed that are located on centres of inversion. The Mn cations are octa­hedrally coordinated by four pyridine coligands and two seleno­cyanate anions that coordinate *via* the N atom to the metal centres to generate *trans*-MnN(s)_2_N(p)_4_ octa­hedra (s = seleno­cyanate and p = pyridine). In the extended structure, weak C—H⋯Se contacts are observed. Powder X-ray diffraction (PXRD) investigations prove that a pure sample was obtained and in the IR and Raman spectra, the C—N stretching vibrations are observed at 2058 and 2060 cm^−1^, respectively, in agreement with the terminal coordination of the seleno­cyanate anions. Thermogravimetric investigations reveal that the pyridine coligands are removed in two separate steps. In the first mass loss, a compound with the composition Mn(NCSe)_2_(C_5_H_5_N)_2_ is formed, whereas in the second mass loss, the remaining pyridine ligands are removed, which is superimposed with the decomposition of Mn(NCSe)_2_ formed after ligand removal. In the inter­mediate compound Mn(NCSe)_2_(C_5_H_5_N)_2_, the CN stretching vibration is observed at 2090 cm^−1^ in the Raman and at 2099 cm^−1^ in the IR spectra, indicating that the Mn cations are linked by μ-1,3-bridging anionic ligands. PXRD measurements show that a compound has formed that is of poor crystallinity. A comparison of the powder pattern with that calculated for the previously reported Cd(NCSe)_2_(C_5_H_5_N)_2_ indicates that these compounds are isotypic, which was proven by a Pawley fit.

## Abstract 2

In the crystal structure of the title compound, discrete complexes are observed, in which the Ni cations are octa­hedrally coordinated by two terminal N-bonded seleno­cyanate anions and four pyridine coligands. Weak C—H⋯Se inter­actions occur in the extended structure.

## 1. Chemical context

Coordination compounds based on transition-metal thio- and seleno­cyanates can be divided into two major groups. In the first group, the anionic ligands are only terminally coordinated, which leads with monocoordinating ligands to discrete complexes that are of inter­est, for example, in the field of spin-crossover materials (Gütlich *et al.*, 2000 ▸; Senthil Kumar & Ruben, 2017 ▸). In the second group, the anionic ligands act as bridging ligands, which is of structural inter­est, because a variety of structures with one-, two- or three-dimensional networks can form. Moreover, because these ligands can mediate magnetic exchange, they are also of inter­est in the field of mol­ecular magnetism (Shurdha *et al.*, 2013 ▸; Palion-Gazda *et al.*, 2015 ▸; Prananto *et al.*, 2017 ▸; Mekuimemba *et al.*, 2018 ▸). In this context, compounds based on Co^II^ cations are of special inter­est because of the strong magnetic anisotropy (Werner *et al.*, 2014 ▸; Rams *et al.*, 2020*a* ▸,*b* ▸; Mautner *et al.*, 2018 ▸).

In the majority of cases, the synthesis of thio- and seleno­cyanate coordination compounds is performed in solution, that with less chalcophilic metal cations frequently leads to the formation of compounds with terminal anionic ligands. In contrast, the synthesis of compounds with a bridging coordination is more difficult to achieve, even if an excess of the metal salt is used in the synthesis. This behaviour is even more pronounced for seleno­cyanate compounds. In such cases, an alternative approach was developed, in which precursor complexes with terminal thio- or seleno­cyanate anions are thermally decomposed, leading to the removal of the coligands in separate steps (Wriedt & Näther, 2010 ▸; Wöhlert *et al.*, 2012 ▸). In the course of this irreversible reaction, the desired compounds with bridging anionic ligands are obtained in qu­anti­tive yields. This approach is of special inter­est for the synthesis of seleno­cyanate coordination polymers because, on one hand, they are frequently difficult to prepare and, on the other hand, only a few such compounds with paramagnetic metal cations are reported in the literature. This method, how­ever, can always be successfully applied for the synthesis of thio­cyanates, whereas for seleno­cyanates sometimes the thermogravimetric (TG) curves are poorly resolved and in some cases poorly crystalline or even amorphous residues are obtained; the reason for this behavior is unknown (Wriedt & Näther, 2010 ▸). 
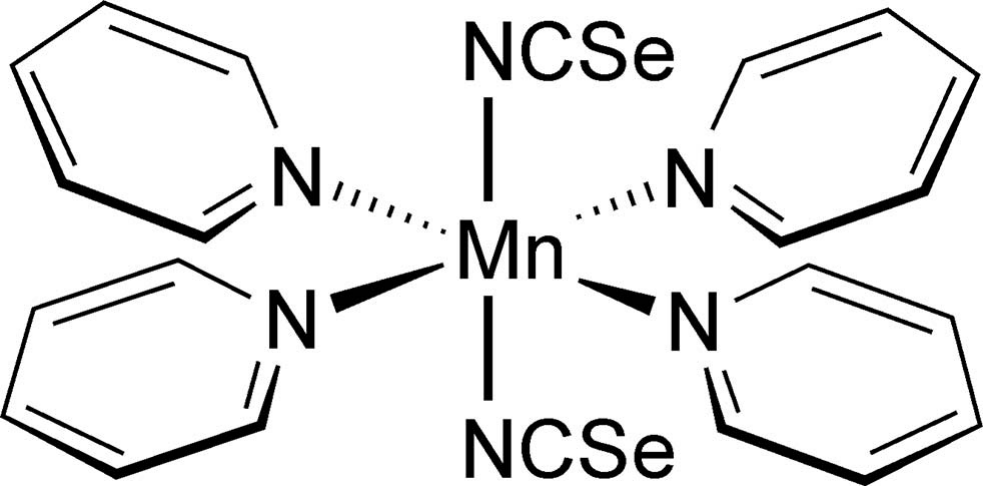


This is the case, for example, for compounds with the composition *M*(NCSe)_2_(C_5_H_5_N)_2_, with *M* = Fe, Co or Ni (C_5_H_5_N is pyridine). The Fe and Co compounds are isotypic to their thio­cyanate analogs and consist of octa­hedrally coordinated metal cations that are linked by pairs of μ-1,3-bridging anionic ligands into chains (Boeckmann & Näther, 2011 ▸; Boeckmann *et al.*, 2012 ▸). None of these compounds can be prepared from solution but the Fe compound is obtained as a pure crystalline material by thermal decomposition of its precursor, whereas the residue obtained for Co is amorphous. However, the Co compound can be prepared in crystalline form by thermal annealing below the decomposition tem­per­ature obtained from TG measurements (Boeckmann & Näther, 2011 ▸). The Ni compound is also available by thermogravimetry, even if it is of low crystallinity, but comparison of the experimental powder X-ray diffraction (PXRD) pattern obtained after the first mass loss shows that Ni(NCSe)_2_(C_5_H_5_N)_2_ is not isotypic to *M*(NCSe)_2_(C_5_H_5_N)_2_, with *M* = Fe, Co and Cd (Näther & Boeckmann, 2023 ▸). Its crystal structure is still unknown and in this context it is mentioned that the occurence of different modifications, including polymorphs or isomers, is frequently observed for such thio­cyanate coordination compounds (Werner *et al.*, 2015 ▸). However, in this case, the question arose if the Mn compound is also available as a crystalline material and if it will adopt the structure type of its Fe, Co and Cd analogs or that of Ni(NCSe)_2_(C_5_H_5_N)_2_.

**Figure 1:**
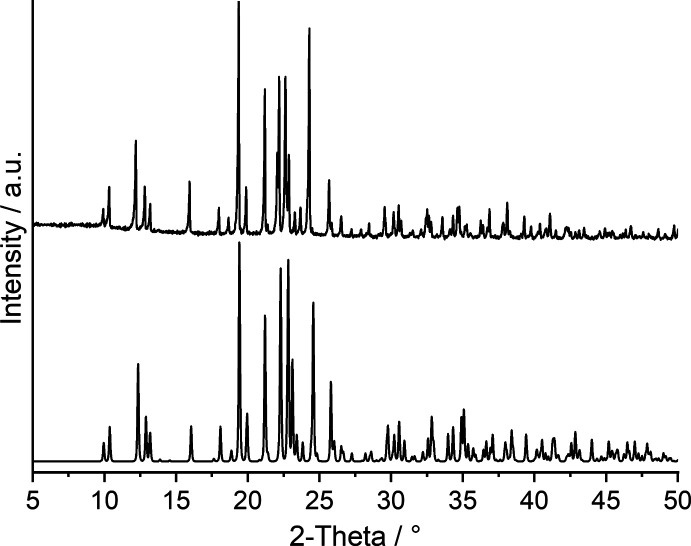
Experimental (top) and calculated PXRD patterns (bottom) of the title compound.

To answer this question, much effort was made to synthesize the desired compound Mn(NCSe)_2_(C_5_H_5_N)_2_ in solution, but the pyridine-rich title compound Mn(NCSe)_2_(C_5_H_5_N)_4_ was always obtained. Single-crystal X-ray diffraction proved that it is isotypic to its Cd, Zn, Fe, Co and Ni analogs (Boeckmann *et al.*, 2011 ▸, 2012 ▸; Boeckmann & Näther, 2011 ▸; Näther & Boeckmann, 2023 ▸) and comparison of the experimental PXRD pattern with that calculated from the single-crystal data proves that a pure phase was obtained (Fig. 1 ▸). Therefore, this compound was used as a precursor for TG investigations to check if the pyridine-deficient compound Mn(NCSe)_2_(C_5_H_5_N)_2_ can be prepared and if this compound is isotypic to its Cd, Fe and Co analogs (see Section 4, *Thermoanalytical investigations*).

## 2. Structural commentary

**Figure 2:**
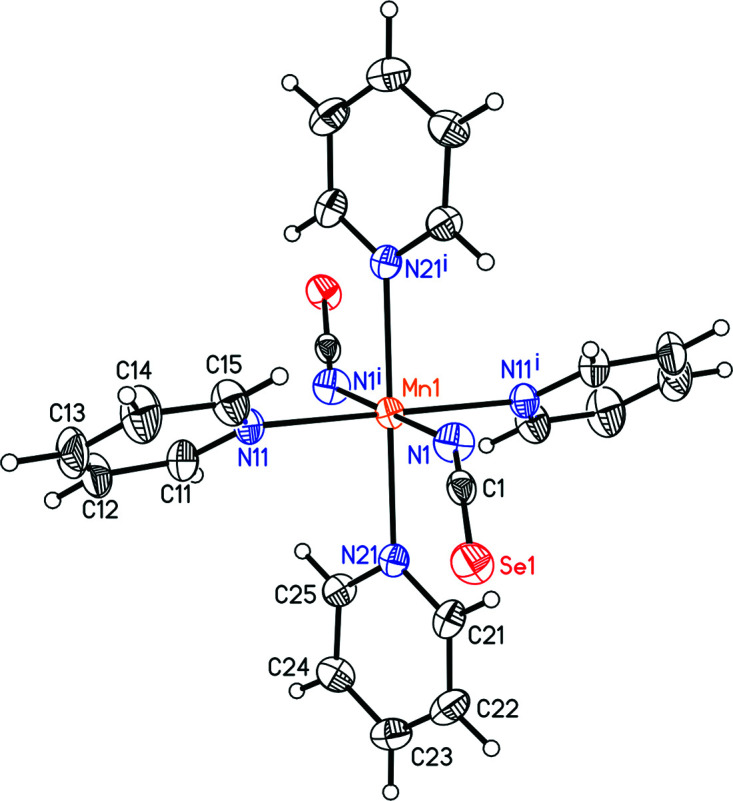
The crystal structure of the title compound, showing the atom labelling and with displacement ellipsoids drawn at the 50% probability level. [Symmetry code: (i) −*x* + 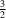 , −*y* + 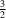 , −*z* + 1.]

The single-crystal structure determination proves that the title compound, Mn(NCSe)_2_(C_5_H_5_N)_4_, consists of discrete com­plexes. The asymmetric unit consists of one crystallo­graphi­cally independent Mn^2+^ cation that is located on a centre of inversion, as well as one seleno­cyanate anion and two pyridine ligands in general positions. The Mn^2+^ cation is octa­hedrally coordinated by four pyridine coligands and two terminally N-bonded thio­cyanate anions that are in *trans* positions (Fig. 2 ▸); the Mn—N bonds to the anions are notably shorter than the bonds to the pyridine mol­ecules (Table 1 ▸) and the bond lengths are similar to those in the corresponding Fe and Co compounds. The *cis*-N—Mn—N angles deviate from the ideal octa­hedral values by up to 2°, which shows that the octa­hedra are slightly distorted.

It is noted that the title compound is isotypic to its Fe, Co, Zn and Cd analogues, with seleno­cyanate ligands, already described in the literature (Boeckmann & Näther, 2011 ▸; Boeckmann *et al.*, 2011 ▸, 2012 ▸). Moreover, the title compound is also isotypic to its thio­cyanate analogs with Cu (Gary *et al.*, 2004 ▸; Li & Zhang, 2004 ▸), Cd (Qu *et al.*, 2004 ▸), Ni (Valach *et al.*, 1984 ▸; Wang *et al.*, 2006 ▸; Małecki *et al.*, 2010 ▸), Fe (Søtofte & Rasmussen, 1967 ▸; Huang & Ogawa, 2006 ▸), Mn (Yang *et al.*, 2007 ▸; Małecki *et al.*, 2011 ▸), Co (Hartl & Brüdgam, 1980 ▸; Li *et al.*, 2007 ▸; Deng *et al.*, 2020 ▸), Mg (Lipkowski & Soldatov, 1993 ▸), Zn (Wu, 2004 ▸) and Co (Neumann *et al.*, 2019 ▸) (see Section 5, *Database survey*).

## 3. Supra­molecular features

**Figure 3:**
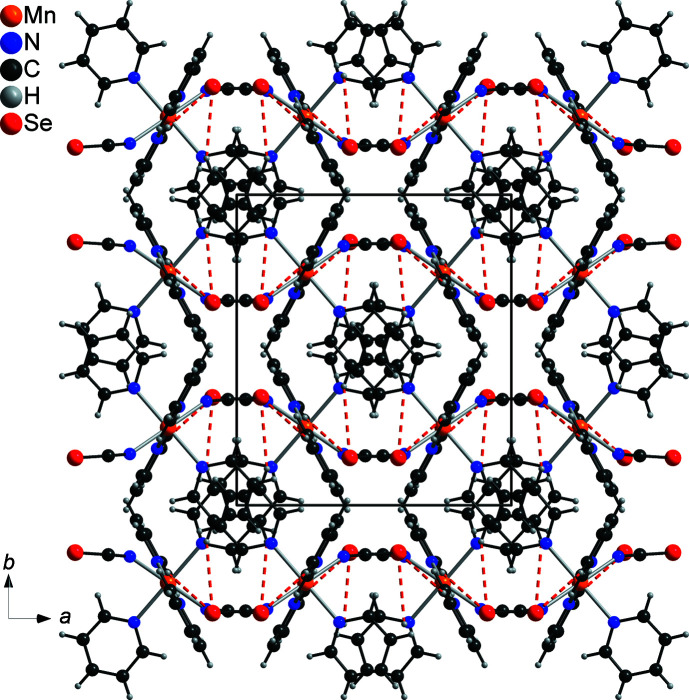
The crystal structure of the title compound, viewed along the crystallographic *c*-axis direction. C—H⋯Se inter­actions are shown as red dashed lines.

In the crystal structure of the title compound, the complexes are arranged into columns that propagate along the crystallographic *c*-axis direction (Fig. 3 ▸). As in the analogous compounds, there is no indication of aromatic π–π stacking inter­actions. Within the structure, the Co(NCSe)_2_ units form corrugated layers that lie parallel to the the *ac* plane (Fig. 3 ▸). Two C—H⋯Se contacts are observed with C—H⋯Se angles above 150°, which might indicate weak hydrogen-bonding inter­actions (Table 2 ▸). There are additional C—H⋯Se and C—H⋯N contacts, but from the distances and angles, it is concluded that they do not correspond to any significant inter­action.

## 4. Thermoanalytical investigations

**Figure 4:**
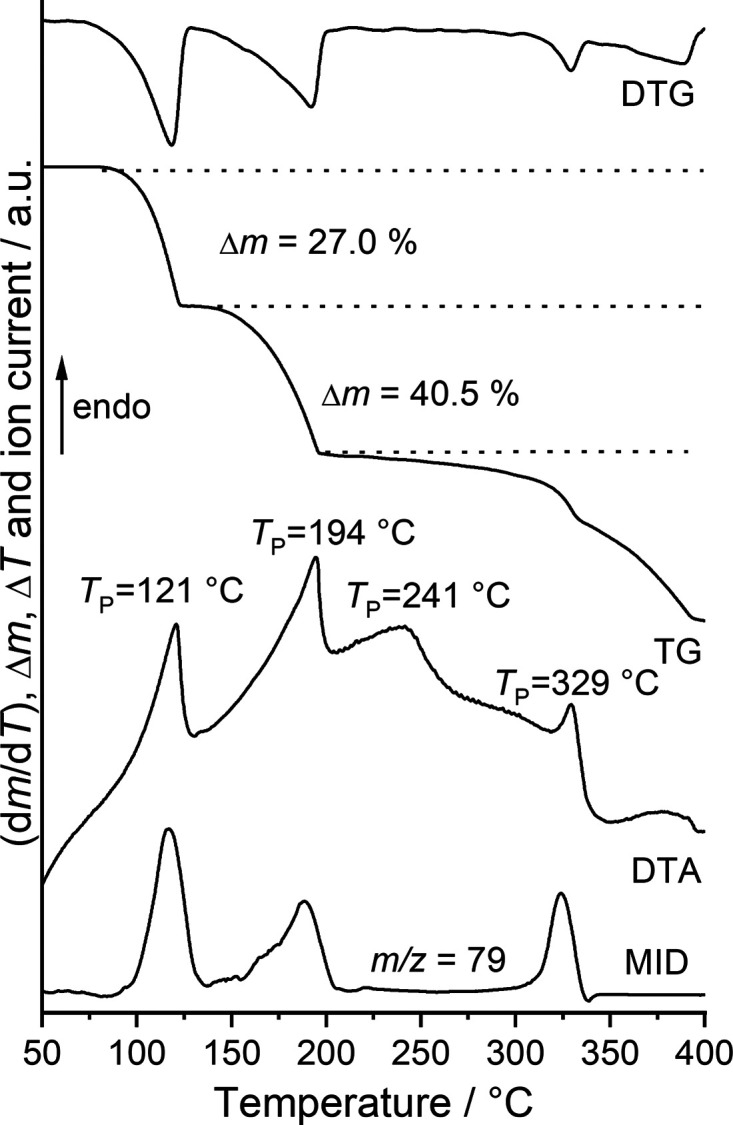
DTG, TG and DTA curves, and the MS trend scan curve for the title compound measured at 4 °C min^−1^ in helium. The experimental mass loss is given in % and the peak temperatures are given in °C.

To investigate the thermal properties of the title compound, measurements using simultaneous differential thermoanalysis and thermogravimetry coupled to mass spectrometry (DTA–TG–MS) were performed. Upon heating, three mass losses are observed that are accompanied by endothermic events in the DTA curve (Fig. 4 ▸). The derivative thermogravimetric (DTG) curve shows that these events are reasonably resolved and from the MS trend scan curve it is obvious that in the first endothermic event, pyridine is removed. The experimental mass loss of 27.0% is in good agreement with that calculated for the removal of two pyridine ligands (Δ*m*_calc_ = −27.2%), whereas that in the second step is higher with a value of 40.5%, indicating that pyridine removal and the decomposition of Mn(NCSe)_2_ occur simultaneously. The fact that even in the third step pyridine might be removed indicates that the reaction is more complex, except that the signal at *m*/*z* = 79 corresponds to some fragment formed during decomposition.

**Figure 5:**
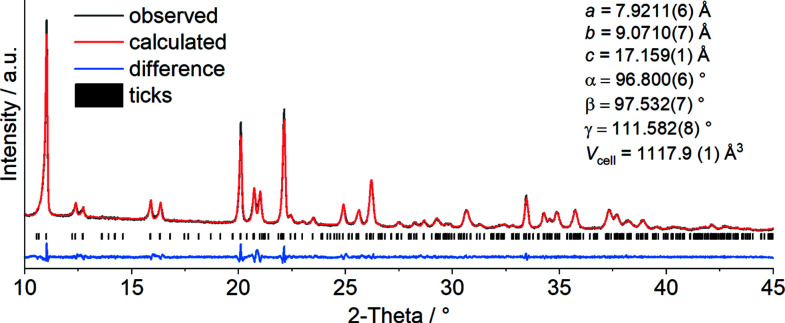
Pawley fit of Mn(NCSe)_2_(pyridine)_2_ obtained by TG measurements of the title compound Mn(NCSe)_2_(pyridine)_4_.

However, to identify the product formed in the first mass loss this residue was isolated in a second TG measurement and investigated by IR and Raman spectroscopy, as well as PXRD. The CN stretching vibration of the seleno­cyanate anions is observed at 2099 cm^−1^ in the Raman and at 2090 cm^−1^ in the IR spectra, clearly proving that μ-1,3-bridging anionic ligands are present (Fig. S2 in the supporting information). The comparison of the experimental pattern with that calculated for Cd(NCSe)_2_(C_5_H_5_N)_2_, using single-crystal data retrieved from the literature, indicate that they are isotypic (Fig. S3). The pattern can easily be indexed, leading to a unit cell that is comparable to that of the Cd, Fe and Co compounds. Finally, a Pawley fit of the experimental pattern using the crystallographic data of the cadmium compound (with Mn replacing Cd) as starting model was performed, which supports all these findings. The refined lattice parameters are shown together with the difference plot in Fig. 5 ▸. As expected, due to the smaller ionic radii, the unit-cell volume is smaller for the Mn compound compared to its Cd anologue [1117.9 (1) *versus* 1145.6 (3) Å^3^] (Boeckmann *et al.*, 2011 ▸).

## 5. Database survey

A search in the Cambridge Structural Database (Version 5.43, last update November 2022; Groom *et al.*, 2016 ▸) using *ConQuest* (Bruno *et al.*, 2002 ▸) reveals that some seleno­cyanate compounds with pyridine coligands have been reported in the literature. These comprise discrete complexes with an octa­hedral coordination with the composition *M*(NCSe)_2_(C_5_H_5_N)_4_, with *M* = Fe (CSD refcode CAQVEX; Boeckmann *et al.*, 2012 ▸), Co (ITISOU; Boeckmann & Näther, 2011 ▸), Zn (OWOHUE; Boeckmann *et al.*, 2011 ▸) and Cd (OWOJAM; Boeckmann *et al.*, 2011 ▸). The isotypic Ni compound *M*(NCSe)_2_(pyridine)_4_ was reported recently (Näther & Boeckmann, 2023 ▸). For the Co compound, mixed crystals with the composition Co(NCS)_*x*_(NCSe)_2–*x*_(C_5_H_5_N)_4_ have also been reported (TIXDOW and TIXDOW01; Neumann *et al.*, 2019 ▸).

As mentioned in the *Chemical context* section (Section 1) with pyridine, there also exist isotypic pyridine-deficient com­pounds with the composition *M*(NCSe)_2_(C_5_H_5_N)_2_, with *M* = Fe (CAQVIB; Boeckmann *et al.*, 2012 ▸), Co (ITISUA; Boeckmann & Näther, 2011 ▸), Zn (OWOJEQ; Boeckmann *et al.*, 2011 ▸) and Cd (OWOHOY; Boeckmann *et al.*, 2011 ▸). In the first compounds, *M*(NCSe)_2_ chains are observed, whereas the Zn compound consists of discrete complexes.

One mixed-metal compound with the composition HgSr(NCSe)_4_(C_5_H_5_N)_6_ (CICLOP; Brodersen *et al.*, 1984 ▸), a dinuclear complex with the composition [Fe(NCS)_2_]_2_(C_5_H_5_N)_2_[(3,5-bis­(pyridin-2-yl)pyrazol­yl]_2_ (FIZYEU; Sy *et al.*, 2014 ▸) and a complex with the composition Fe(NCSe)_2_(C_5_H_5_N)_2_(2-methyl­dipyrido[3,2-*f*:2′,3′-*h*]quinoxaline) pyridine solvate (TISWOI; Tao *et al.*, 2007 ▸) are also reported in the literature.

## 6. Synthesis and crystallization

MnCl_2_·2H_2_O, KNCSe and pyridine were purchased from Alfa Aesar and used without any further purification.

### 6.1. Synthesis

0.25 mmol (49.7 mg) MnCl_2_·2H_2_O and 0.5 mmol (72.0 mg) KNCSe were reacted with a mixture of 1.5 ml of pyridine and 1.5 ml of water. The mixture was stirred for 2 d at room temperature and the precipitate was filtered off, washed with very small amounts of water and dried in air. Single crystals were obtained under the same conditions but without stirring.

It is noted that even if a large excess of MnCl_2_·2H_2_O and KNCSe was used in the synthesis, there are no hints for the formation of a pyridine-deficient compound with the composition Mn(NCSe)_2_(pyridine)_2_.

### 6.2. Experimental details

Single-crystal X-ray data were measured using an Image Plate Diffraction System (IPDS-2) from Stoe & Cie. Differential thermal analysis and thermogravimetric (DTA–TG–MS) measurements were performed in a dynamic helium atmosphere in Al_2_O_3_ crucibles using a Netzsch thermobalance with a skimmer coupling and a Balzer Quadrupol MS. The PXRD measurements were performed with a Stoe Transmission Powder Diffraction System (STADI P) with Cu *K*α_1_ radiation equipped with a linear position-sensitive MYTHEN 1K detector from Stoe & Cie. The IR data were measured using a Bruker Alpha-P ATR–IR spectrometer and the Raman spectra were measured with a Bruker Vertex 70 spectrometer.

## 7. Refinement

H atoms were positioned with idealized geometry (C—H = 0.95 Å) and refined with *U*_iso_(H) = 1.2*U*_eq_(C) using a riding model. Crystal data, data collection and structure refinement details are summarized in Table 3 ▸.

The Pawely fit for the diffraction pattern of Mn(NCSe)_2_(C_5_H_5_N)_2_ obtained by thermal decomposition was carried out using *TOPAS Academic* (Version 6.0; Coelho 2018 ▸). Initial lattice parameters were taken from Cd(NCSe)_2_(C_5_H_5_N)_2_: *R*_wp_ = 2.98%, *R*_exp _ = 2.08% and GOF = 1.44.

## Supplementary Material

Crystal structure: contains datablock(s) I, global. DOI: 10.1107/S2056989023003535/hb8063sup1.cif

Structure factors: contains datablock(s) I. DOI: 10.1107/S2056989023003535/hb8063Isup2.hkl

Additional spectra. DOI: 10.1107/S2056989023003535/hb8063sup3.pdf

CCDC reference: 2257160

Additional supporting information:  crystallographic information; 3D view; checkCIF report

## Figures and Tables

**Table 1:** Selected geometric parameters (Å, °)

Mn1—N1	2.196 (3)	Mn1—N21	2.317 (3)
Mn1—N11	2.307 (3)		
			
N1—Mn1—N11^i^	90.59 (10)	N11—Mn1—N21	92.03 (9)
N1—Mn1—N11	89.41 (10)	N11—Mn1—N21^i^	87.97 (9)
N1^i^—Mn1—N21	90.02 (10)	N1—C1—Se1	179.1 (3)
N1—Mn1—N21	89.98 (10)	C1—N1—Mn1	151.4 (3)

Symmetry code: (i) 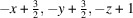 .

**Table 2:** Hydrogen-bond geometry (Å, °)

*D*—H⋯*A*	*D*—H	H⋯*A*	*D*⋯*A*	*D*—H⋯*A*
C12—H12⋯Se1^ii^	0.95	3.10	3.992 (4)	156
C22—H22⋯Se1^iii^	0.95	3.11	3.986 (4)	155
C25—H25⋯Se1^iv^	0.95	3.04	3.757 (3)	133
C25—H25⋯N1^i^	0.95	2.65	3.247 (4)	121

Symmetry codes: (i) 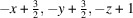 ; (ii) 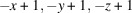 ; (iii) 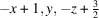 ; (iv) 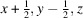 .

**Table 3:** Experimental details

Crystal data	
Chemical formula	[Mn(NCSe)_2_(C_5_H_5_N)_2_]
*M*_r_	581.30
Crystal system, space group	Monoclinic, *C*2/*c*
Temperature (K)	170
*a*, *b*, *c* (Å)	12.5335 (9), 13.4331 (8), 15.1300 (12)
β (°)	108.608 (8)
*V* (Å^3^)	2414.2 (3)
*Z*	4
Radiation type	Mo *K*α
μ (mm^−1^)	3.58
Crystal size (mm)	0.25 × 0.2 × 0.17

Data collection	
Diffractometer	Stoe IPDS2
Absorption correction	Numerical
*T*_min_, *T*_max_	0.332, 0.678
No. of measured, independent and observed [*I* > 2σ(*I*)] reflections	8352, 2636, 2124
*R*_int_	0.056
(sin θ/λ)_max_ (Å^−1^)	0.639

Refinement	
*R*[*F* ^2^ > 2σ(*F* ^2^)], *wR*(*F* ^2^), *S*	0.043, 0.088, 1.03
No. of reflections	2636
No. of parameters	142
H-atom treatment	H-atom parameters constrained
Δρ_max_, Δρ_min_ (e Å^−3^)	0.56, −0.59

Computer programs: *X-AREA* (Stoe & Cie, 2008 ▸), *SHELXT2014* (Sheldrick, 2015*a* ▸), *SHELXL2016* (Sheldrick, 2015*b* ▸), *DIAMOND* (Brandenburg & Putz, 1999 ▸) and *publCIF* (Westrip, 2010 ▸).

## supplementary crystallographic information

### Crystal data

**Table:**

[Mn(NCSe)_2_(C_5_H_5_N)_2_]	*F*(000) = 1148
*M**_r_* = 581.30	*D*_x_ = 1.599 Mg m^−^^3^
Monoclinic, *C*2/*c*	Mo *K*α radiation, λ = 0.71073 Å
*a* = 12.5335 (9) Å	Cell parameters from 8352 reflections
*b* = 13.4331 (8) Å	θ = 2.4–27.0°
*c* = 15.1300 (12) Å	µ = 3.58 mm^−^^1^
β = 108.608 (8)°	*T* = 170 K
*V* = 2414.2 (3) Å^3^	Block, colorless
*Z* = 4	0.25 × 0.2 × 0.17 mm

### Data collection

**Table:**

Stoe IPDS-2 diffractometer	2124 reflections with *I* > 2σ(*I*)
ω scans	*R*_int_ = 0.056
Absorption correction: numerical	θ_max_ = 27.0°, θ_min_ = 2.4°
*T*_min_ = 0.332, *T*_max_ = 0.678	*h* = −16→16
8352 measured reflections	*k* = −17→16
2636 independent reflections	*l* = −18→19

### Refinement

**Table:**

Refinement on *F*^2^	0 restraints
Least-squares matrix: full	Hydrogen site location: inferred from neighbouring sites
*R*[*F*^2^ > 2σ(*F*^2^)] = 0.043	H-atom parameters constrained
*wR*(*F*^2^) = 0.088	*w* = 1/[σ^2^(*F*_o_^2^) + (0.0376*P*)^2^ + 6.3344*P*] where *P* = (*F*_o_^2^ + 2*F*_c_^2^)/3
*S* = 1.03	(Δ/σ)_max_ = 0.001
2636 reflections	Δρ_max_ = 0.56 e Å^−^^3^
142 parameters	Δρ_min_ = −0.59 e Å^−^^3^

### Special details

**Table:**

Geometry. All esds (except the esd in the dihedral angle between two l.s. planes) are estimated using the full covariance matrix. The cell esds are taken into account individually in the estimation of esds in distances, angles and torsion angles; correlations between esds in cell parameters are only used when they are defined by crystal symmetry. An approximate (isotropic) treatment of cell esds is used for estimating esds involving l.s. planes.

### Fractional atomic coordinates and isotropic or equivalent isotropic displacement parameters (Å^2^)

**Table:**

	*x*	*y*	*z*	*U*_iso_*/*U*_eq_	
Mn1	0.750000	0.750000	0.500000	0.01953 (15)	
N11	0.6262 (2)	0.6231 (2)	0.43418 (18)	0.0238 (6)	
C11	0.6575 (3)	0.5279 (3)	0.4461 (2)	0.0318 (8)	
H11	0.734043	0.513251	0.479058	0.038*	
C12	0.5845 (3)	0.4496 (3)	0.4129 (3)	0.0406 (9)	
H12	0.610081	0.382797	0.423720	0.049*	
C13	0.4740 (3)	0.4701 (3)	0.3639 (3)	0.0454 (10)	
H13	0.421823	0.417644	0.340179	0.054*	
C14	0.4403 (3)	0.5677 (3)	0.3500 (3)	0.0480 (11)	
H14	0.364597	0.583892	0.315879	0.058*	
C15	0.5183 (3)	0.6422 (3)	0.3864 (3)	0.0347 (8)	
H15	0.494330	0.709602	0.376995	0.042*	
N21	0.7901 (2)	0.6823 (2)	0.64791 (18)	0.0222 (5)	
C21	0.7567 (3)	0.7256 (3)	0.7149 (2)	0.0284 (7)	
H21	0.717815	0.787259	0.701489	0.034*	
C22	0.7765 (3)	0.6842 (3)	0.8025 (2)	0.0356 (8)	
H22	0.752223	0.717571	0.848060	0.043*	
C23	0.8319 (3)	0.5938 (3)	0.8230 (2)	0.0322 (8)	
H23	0.846555	0.564051	0.882662	0.039*	
C24	0.8654 (3)	0.5481 (3)	0.7544 (2)	0.0303 (7)	
H24	0.902487	0.485410	0.765582	0.036*	
C25	0.8441 (3)	0.5951 (3)	0.6690 (2)	0.0268 (7)	
H25	0.869203	0.563732	0.622887	0.032*	
Se1	0.41161 (3)	0.84748 (3)	0.58422 (3)	0.03339 (12)	
C1	0.5323 (3)	0.8406 (2)	0.5460 (2)	0.0220 (6)	
N1	0.6105 (2)	0.8349 (2)	0.5213 (2)	0.0291 (6)	

### Atomic displacement parameters (Å^2^)

**Table:**

	*U*^11^	*U*^22^	*U*^33^	*U*^12^	*U*^13^	*U*^23^
Mn1	0.0146 (3)	0.0198 (3)	0.0244 (3)	0.0001 (2)	0.0065 (2)	−0.0004 (3)
N11	0.0202 (12)	0.0227 (14)	0.0273 (14)	−0.0022 (10)	0.0058 (10)	−0.0023 (11)
C11	0.0340 (18)	0.0243 (18)	0.0366 (19)	−0.0002 (14)	0.0106 (15)	−0.0059 (14)
C12	0.054 (2)	0.0227 (18)	0.048 (2)	−0.0072 (18)	0.0202 (18)	−0.0072 (17)
C13	0.042 (2)	0.042 (2)	0.054 (2)	−0.0259 (19)	0.0172 (18)	−0.0170 (19)
C14	0.0282 (18)	0.047 (3)	0.059 (3)	−0.0131 (18)	−0.0003 (18)	−0.008 (2)
C15	0.0248 (16)	0.0302 (19)	0.043 (2)	−0.0020 (15)	0.0023 (15)	0.0006 (16)
N21	0.0169 (12)	0.0243 (14)	0.0243 (13)	−0.0015 (10)	0.0050 (10)	−0.0021 (10)
C21	0.0251 (15)	0.0305 (18)	0.0289 (17)	0.0007 (13)	0.0077 (13)	−0.0075 (13)
C22	0.0363 (19)	0.047 (2)	0.0261 (17)	−0.0025 (17)	0.0132 (14)	−0.0077 (15)
C23	0.0258 (16)	0.045 (2)	0.0255 (17)	−0.0024 (15)	0.0076 (13)	0.0036 (15)
C24	0.0242 (15)	0.0310 (18)	0.0338 (17)	0.0053 (14)	0.0066 (13)	0.0075 (15)
C25	0.0263 (16)	0.0279 (18)	0.0257 (16)	0.0040 (13)	0.0077 (13)	0.0007 (12)
Se1	0.0337 (2)	0.0309 (2)	0.0452 (2)	0.00150 (15)	0.02617 (16)	−0.00001 (16)
C1	0.0236 (14)	0.0158 (14)	0.0221 (15)	0.0007 (12)	0.0010 (12)	0.0007 (12)
N1	0.0211 (13)	0.0334 (16)	0.0351 (15)	0.0070 (12)	0.0120 (11)	0.0015 (12)

### Geometric parameters (Å, º)

**Table:**

Mn1—N1^i^	2.196 (3)	C14—H14	0.9500
Mn1—N1	2.196 (3)	C15—H15	0.9500
Mn1—N11^i^	2.307 (3)	N21—C25	1.340 (4)
Mn1—N11	2.307 (3)	N21—C21	1.346 (4)
Mn1—N21	2.317 (3)	C21—C22	1.384 (5)
Mn1—N21^i^	2.317 (3)	C21—H21	0.9500
N11—C11	1.332 (4)	C22—C23	1.384 (5)
N11—C15	1.339 (4)	C22—H22	0.9500
C11—C12	1.379 (5)	C23—C24	1.383 (5)
C11—H11	0.9500	C23—H23	0.9500
C12—C13	1.374 (6)	C24—C25	1.385 (5)
C12—H12	0.9500	C24—H24	0.9500
C13—C14	1.373 (6)	C25—H25	0.9500
C13—H13	0.9500	Se1—C1	1.786 (3)
C14—C15	1.384 (5)	C1—N1	1.157 (4)
			
N1^i^—Mn1—N1	180.00 (15)	C13—C14—C15	119.1 (4)
N1^i^—Mn1—N11^i^	89.41 (10)	C13—C14—H14	120.4
N1—Mn1—N11^i^	90.59 (10)	C15—C14—H14	120.4
N1^i^—Mn1—N11	90.59 (10)	N11—C15—C14	122.6 (4)
N1—Mn1—N11	89.41 (10)	N11—C15—H15	118.7
N11^i^—Mn1—N11	180.0	C14—C15—H15	118.7
N1^i^—Mn1—N21	90.02 (10)	C25—N21—C21	117.0 (3)
N1—Mn1—N21	89.98 (10)	C25—N21—Mn1	120.7 (2)
N11^i^—Mn1—N21	87.97 (9)	C21—N21—Mn1	122.3 (2)
N11—Mn1—N21	92.03 (9)	N21—C21—C22	122.9 (3)
N1^i^—Mn1—N21^i^	89.98 (10)	N21—C21—H21	118.5
N1—Mn1—N21^i^	90.02 (10)	C22—C21—H21	118.5
N11^i^—Mn1—N21^i^	92.03 (9)	C21—C22—C23	119.4 (3)
N11—Mn1—N21^i^	87.97 (9)	C21—C22—H22	120.3
N21—Mn1—N21^i^	180.0	C23—C22—H22	120.3
C11—N11—C15	117.4 (3)	C24—C23—C22	118.2 (3)
C11—N11—Mn1	121.5 (2)	C24—C23—H23	120.9
C15—N11—Mn1	121.0 (2)	C22—C23—H23	120.9
N11—C11—C12	123.3 (3)	C23—C24—C25	118.9 (3)
N11—C11—H11	118.3	C23—C24—H24	120.5
C12—C11—H11	118.3	C25—C24—H24	120.5
C13—C12—C11	118.8 (4)	N21—C25—C24	123.6 (3)
C13—C12—H12	120.6	N21—C25—H25	118.2
C11—C12—H12	120.6	C24—C25—H25	118.2
C14—C13—C12	118.7 (3)	N1—C1—Se1	179.1 (3)
C14—C13—H13	120.6	C1—N1—Mn1	151.4 (3)
C12—C13—H13	120.6		

Symmetry code: (i) −*x*+3/2, −*y*+3/2, −*z*+1.

### Hydrogen-bond geometry (Å, º)

**Table:**

*D*—H···*A*	*D*—H	H···*A*	*D*···*A*	*D*—H···*A*
C12—H12···Se1^ii^	0.95	3.10	3.992 (4)	156
C22—H22···Se1^iii^	0.95	3.11	3.986 (4)	155
C25—H25···Se1^iv^	0.95	3.04	3.757 (3)	133
C25—H25···N1^i^	0.95	2.65	3.247 (4)	121

Symmetry codes: (i) −*x*+3/2, −*y*+3/2, −*z*+1; (ii) −*x*+1, −*y*+1, −*z*+1; (iii) −*x*+1, *y*, −*z*+3/2; (iv) *x*+1/2, *y*−1/2, *z*.
