# Supplementary material for: Synthesis, crystal structure and thermal decomposition pathway of bis­(iso­seleno­cyanato-κN)tetra­kis­(pyridine-κN)manganese(II)
Source: Acta Crystallogr E Crystallogr Commun. 2023 Apr 21;79(Pt 5):482–7. doi: 10.1107/S2056989023003535 (PMC10162072; doi:10.1107/S2056989023003535)
Supplement: Supplementary file 3 [file e-79-00482-sup3.pdf]

STRUCTURE REPORTS

Volume 79 (2023)

Supporting information for article:

Synthesis, crystal structure and thermal decomposition pathway of bis-(isoselenocyanato-κ*N*)tetrakis(pyridine-κ*N*)manganese(II)

Christian Näther, Sebastian Mangelsen and Jan Boeckmann

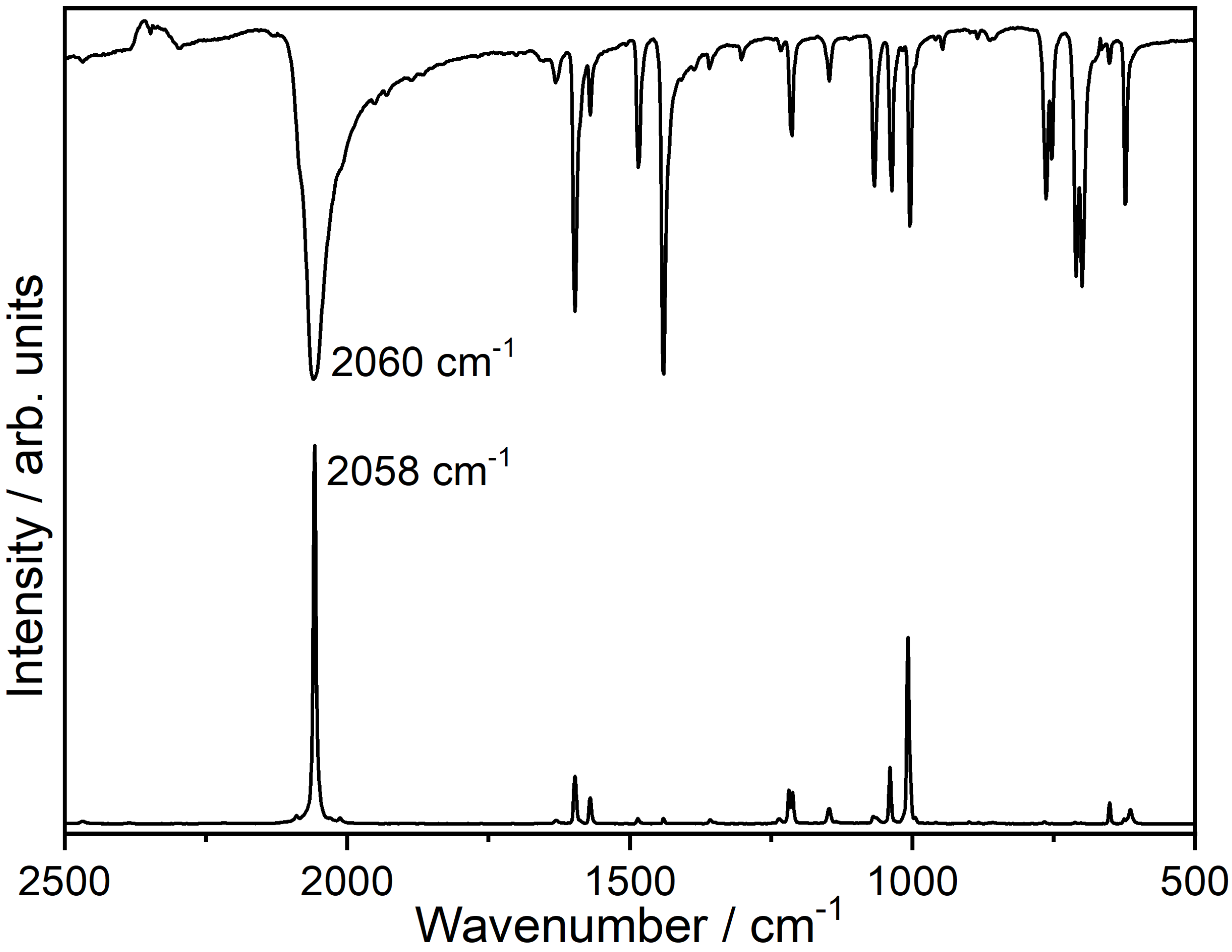

Fig. S1.

IR (top) and Raman spectra (bottom) of the residue obtained after the first mass loss in a TG measurement of the title compound. Given are the values for the CN stretching vibrations of the selenocyanate anion.

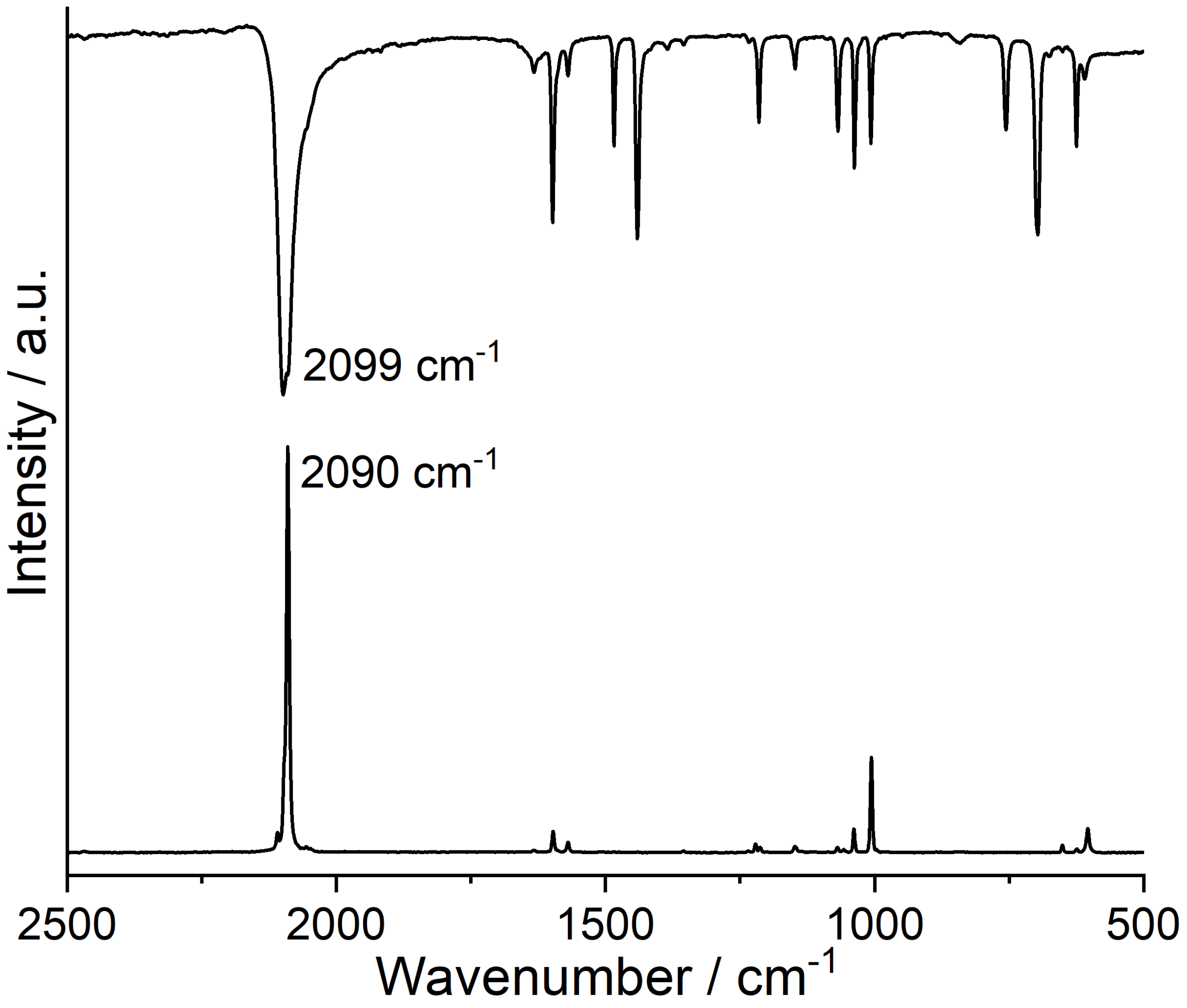

Fig. S2.

Experimental PXRD pattern of the residue obtained after the first mass loss in a TG measurement of the title compound (top) and calculated PXRD pattern for $Cd(NCSe)_2(pyridine)_2$ retrieved from literature (Boeckmann *et al*., 2011, bottom).

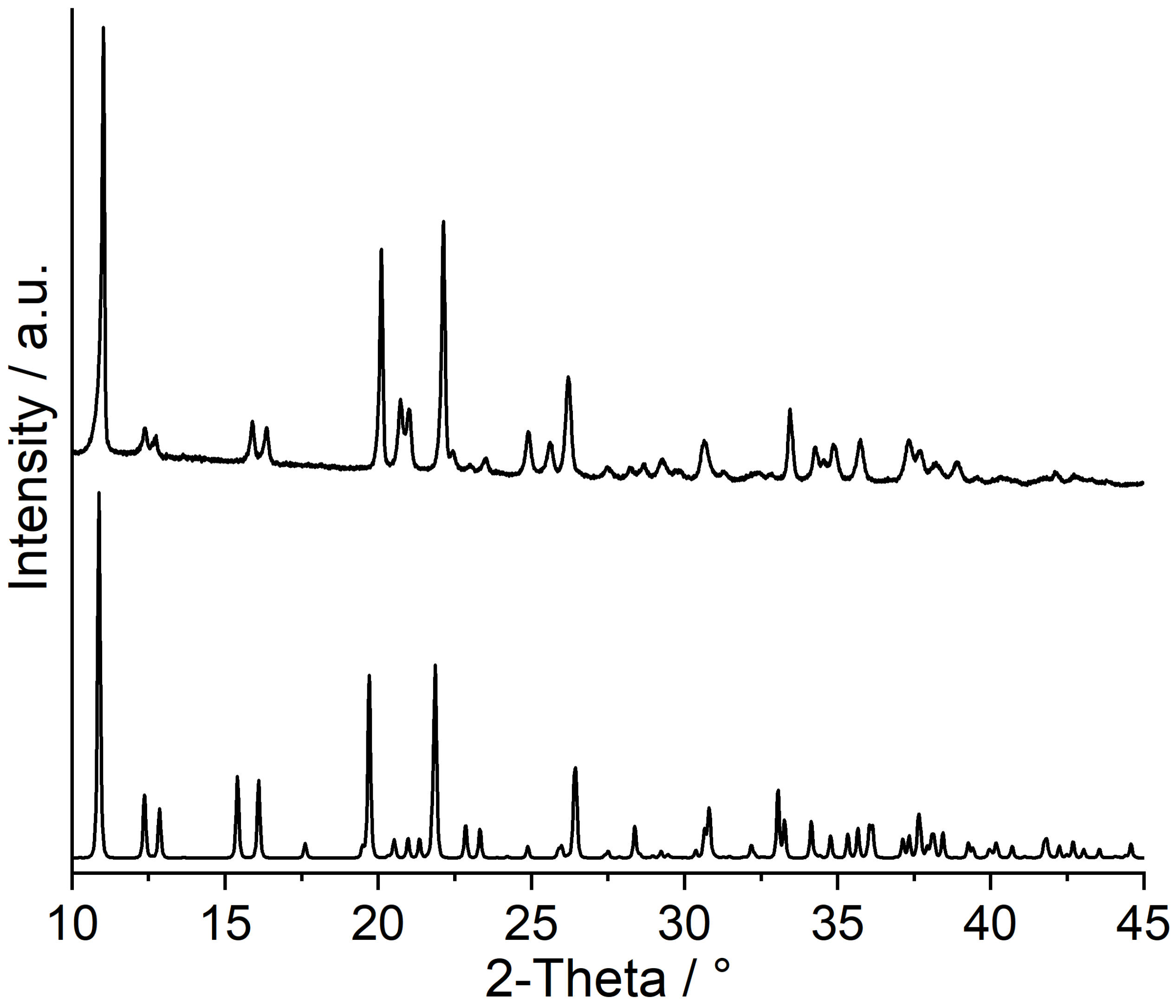

Fig. S3.

In the crystal structure of the title compound, discrete complexes are observed, in which the Ni cations are octahedrally coordinated by two terminal N-bonded seleno-cyanate anions and four pyridine coligands. Weak C-H...Se interactions occur in the extended structure.
